# Light-activated antimicrobial coatings: the great potential of organic photosensitizers

**DOI:** 10.1039/d5ra00272a

**Published:** 2025-03-13

**Authors:** Karolina Socha, Ivan Gusev, Patryk Mroczko, Agata Blacha-Grzechnik

**Affiliations:** a Silesian University of Technology, Faculty of Chemistry Strzody 9 Gliwice 44-100 Poland agata.blacha@polsl.pl; b Silesian University of Technology, Centre for Organic and Nanohybrid Electronics Konarskiego 22B Gliwice 44-100 Poland

## Abstract

Contamination of inanimate surfaces with microorganisms is considered one of the routes for transmission of pathogens, which is a matter of concern not only in healthcare-related facilities, but also in public areas. Durable antimicrobial coatings have emerged as the one of most promising strategies for reducing the accumulation of microorganisms on high-touch surfaces. Light-activated antimicrobial layers are of particular interest for such a purpose, as they generate singlet oxygen and other reactive oxygen species that are effective against a broad spectrum of bacteria, viruses, and fungi. In this review, the antimicrobial coatings containing organic photosensitizers are discussed, focusing on the recent advances in the strategies for PSs' immobilization on solid surfaces. The review attempts to assess the advantages and limitations of those systems, and the challenges that still need to be overcome.

## Abstract 2

Durable antimicrobial coatings has emerged as the one of most promising strategies for reducing the accumulation of microorganisms on high-touch surfaces. In this group, light-activated layers are of particular interest, as they are effective against broad range of pathogens.

## Graphical abstract

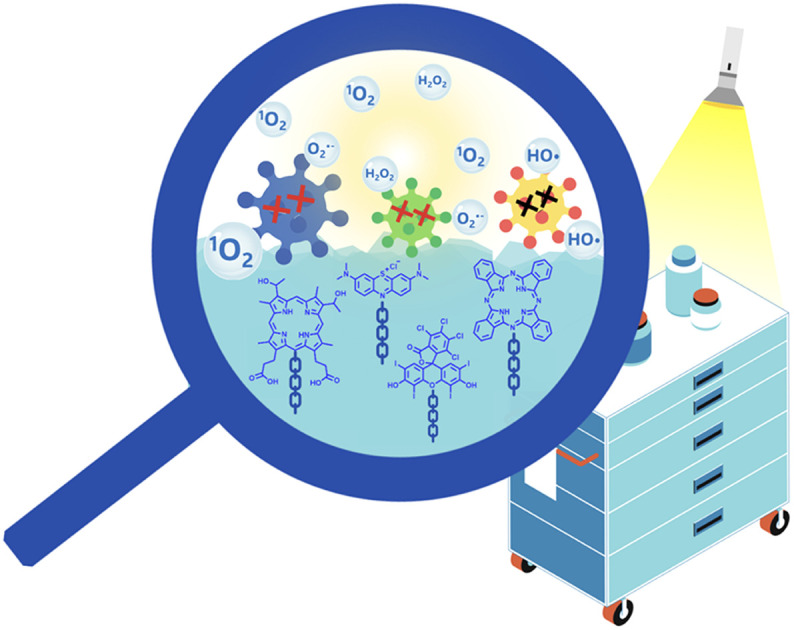

## Introduction

Hospital acquired infections (HAI, nosocomial infections) remain one of the greatest challenges for healthcare systems worldwide. In Europe and the Western Pacific, the hospital-acquired infection rate is between 7.7% and 9%, *ca.* 11% in the Middle East, and *ca.* 10% in Southeast Asia. In some countries, the rate reaches up to 20%.^1^ HAIs result in prolonged treatment, increased number of lethal cases, and higher costs of treatment.^1,2^ Several routes of transmission of HAI pathogens have been identified, including direct transmission between patients/workers, or indirect routes *via* contaminated medical devices or high-touch surfaces.^3,4^ While it has been agreed that proper environmental and hand hygiene or use of protective equipment can significantly reduce HAI case rate, the suitable treatment of inanimate surfaces contaminated with microorganisms has been under longer debate.^2,5,6^ Moreover, the recent pandemic caused by SARS-CoV-2 showed that the presence of pathogens is also problematic for high-touch surfaces in non-healthcare-related public areas.

Depending on the reduction level, the following terms are used for the microorganisms' inactivation: cleaning, sanitizing, disinfecting, and sterilizing, with the last one yielding complete removal of all forms of microbes. Mechanical (*e.g.* brushing & water jet), chemical (*e.g.* detergents, oxidizing agents, ionic surfactants, halogenated compounds), and physical (*e.g.* ultrasounds, UV-light, autoclave) methods are commonly applied on different stages of surface treatment. A combination of different techniques is also frequently used. The selection of proper cleaning procedure depends on a type of surface and its role, *e.g.* high-touch surfaces (door knobs, light switches, handrails) in healthcare units or public areas, medical device surfaces, food contact surfaces, *etc.*^2,5^

Various pathogenic microorganisms can persevere on inanimate surfaces for several weeks or even months and can be re-deposited rapidly after disinfection,^2,5,7^ thus the alternative preventive measure, *i.e.* modification of objects' surface with antimicrobial coatings, has been proposed.^7,8^ Such layers covering fabrics, metals, plastics, *etc.* should be active against a broad range of pathogens, easy to fabricate, and safe for end-users. In the area of antimicrobial coatings two groups can be distinguished: (i) anti-fouling and antiadhesive, *i.e.* lowering microorganisms' adhesion (*e.g.* poly(ethylene glycol)) and (ii) active ones, *i.e.* destroying already adhered microorganisms (*e.g.* silver- or copper-containing coatings, polycationic layers).^8^ In particular cases, the antimicrobial effect is connected with a release of an active agent into the environment, *e.g.* coatings with bactericidal ions or antibiotics on implants.^9^ The target use of the material is the main criterion for the selection of the type of antimicrobial coating. However, the types of pathogens, environment, mechanism of action, user safety, or biocompatibility need to be taken into account.

The subject of this review – the light-activated antimicrobial layers, falls into the category of active antimicrobial coatings. The mechanism of action is based on the production of reactive oxygen species (ROS). This type of coating has been under high research interest for many years now.^8,10^ Though, till now mostly inorganic-based coatings have been employed in general use, the ones consisting of organic photosensitizers possess several advantages and thus, are still widely investigated. This review aims to summarize the recent advances in the light-activated antimicrobial organic layers. In the first part, reactive oxygen species, the organic photosensitizers, and the mechanism of antimicrobial action will be introduced. Next, the recent strategies for the formation of light-activated antimicrobial layers will be reviewed. In the final part, the future outlook will be discussed, emphasizing the challenges that still need to be overcome.

## Reactive oxygen species, organic photosensitizers & photodynamic antimicrobial therapy

Reactive oxygen species (ROS) is a group of highly active forms of oxygen that includes superoxide anion radical, hydrogen peroxide, singlet oxygen, and hydroxyl radical.^11^ ROS are produced during normal oxygen metabolism, however, if the amount of ROS is significantly increased, the cell reaches a state of oxidative stress with an impairment of cellular structures. The excessive levels of ROS can cause severe damage to DNA and proteins.^12^

**Fig. 1:**
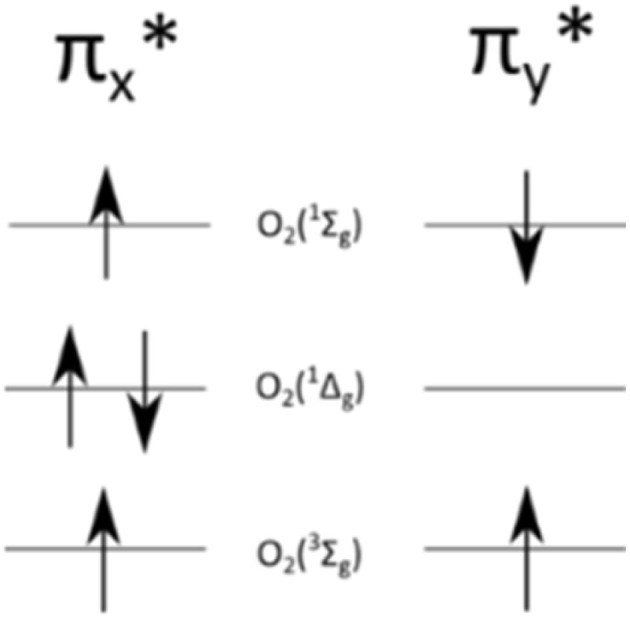
π* orbital for oxygen molecule in various states.

In the group of ROS, singlet oxygen, O_2_(^1^Δ_g_), is the unique molecule. It is the lowest excited state of oxygen molecule, that unlike triplet oxygen, O_2_(^3^Σ_g_^−^), has no unpaired electrons on π* orbital (Fig. 1).^13^ The other singlet form, O_2_(^1^Σ_g_^+^), rapidly decays,^7,8^ thus the term singlet oxygen usually refers to O_2_(^1^Δ_g_).^14,15^^1^O_2_ possess remarkable properties, such as high reactivity and strong oxidizing properties.^16,17^ The lifetime of singlet oxygen lies in the μs–ms range depending on the solvent type, and temperature, interacting quickly with other molecules in the surroundings.^17,18^ Lately, Wang *et al.* estimated that under everyday atmospheric conditions, *i.e.* 23 °C and 1 atm, singlet oxygen's lifetime in air is equal to 2.80 s and it diffuses *ca.* 0.992 cm.^19^

**Fig. 2:**
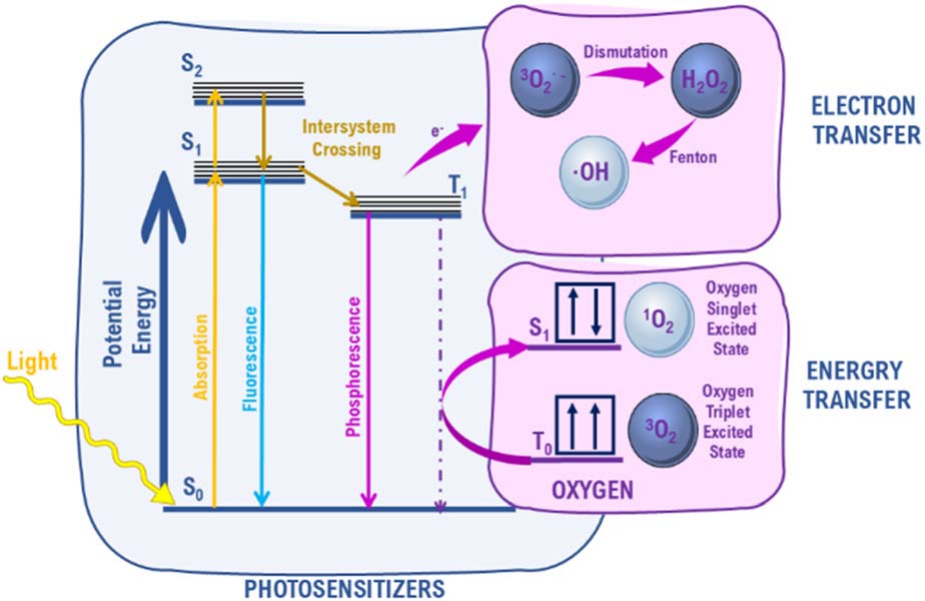
Jablonski diagram of organic photosensitizer.

Photosensitization is one of the most efficient methods of singlet oxygen production. It involves the absorption of light by so called photosensitizer (PS) molecule and a transfer of energy to ground state oxygen. The absorption of energy by organic photosensitizer causes its transition from a ground singlet state, S_0_, to an excited singlet state, *S*_n_ (Fig. 2). This is followed by the non-radiative transition to the first high-energy singlet state, S_1_. The transition from the singlet excited state, S_1_, to a triplet excited state, T_1_, *i.e.* intersystem crossing, is crucial. These are forbidden transitions associated with a change in electron spin. Photosensitizer in the T_1_ state can transfer energy to triplet oxygen yielding singlet oxygen. It is also possible to observe the electron transfer process yielding other types of ROS.^13,17^ It has to be noted that several competitive processes may occur, *e.g.* fluorescence or phosphorescence, that may significantly reduce the yield of ROS production.

Photosensitizers can be generally classified into the following groups:^17^

(1) Organic PSs: phenothiazines,^20–22^ crystal violet,^23^ porphyrins,^24,25^ porphycenes,^26^ phthalocyanines,^27,28^ chlorines,^29^ texaphyrins,^30^ indocyanine dyes,^31,32^ eosin y,^33^ boron-dipyrromethene (BODIPY),^34,35^ diketopyrrolopyrrole,^36,37^ xanthenes,^38–40^ squaraines,^41^ curcuminoids^42,43^ and chalcogenopyrylium dyes.^44,45^

(2) Inorganic PSs: metals and metal oxides such as iridium,^46^ gold,^47^ zinc oxide,^48,49^ and titanium oxide.^50–52^

(3) Heavy metal complexes: ruthenium,^53,54^ iridium,^55,56^ platinum.^57^

It has to be noted that the mechanism of ROS production in the case of inorganic compounds is different. The light absorption causes the generation of electron (e^−^)–hole (h^+^) pair that further undergo redox reactions yielding ROS.^58^

**Fig. 3:**
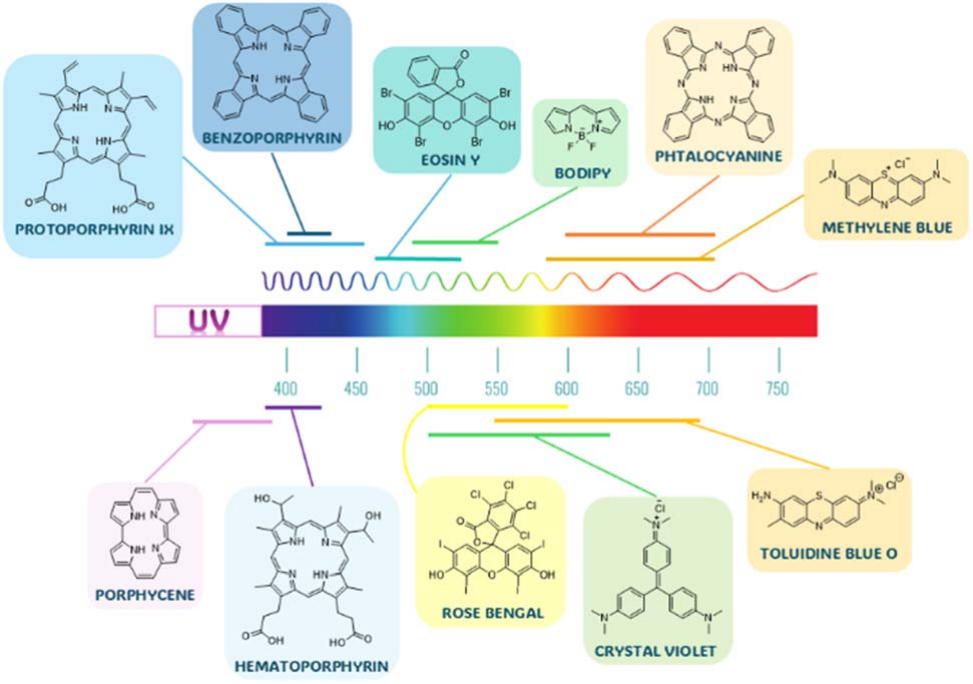
Examples of organic photosensitizers and their corresponding absorption ranges.

Organic and inorganic sources of ROS differ significantly in the absorption range. While inorganic ones absorb mainly in the UV region, organic photosensitizers possess also strong absorption bands in various parts of the visible region (Fig. 3).^17^ This is, of course, particularly advantageous for any visible-light-driven photocatalytic applications. Moreover, the chemical structure of organic PSs can be fine-tuned to control not only their absorption but also the solubility or quantum yield of singlet oxygen production.^59,60^

In the case of antimicrobial action, the electrostatic interaction of PS needs to be taken into account when designing photosensitizer molecules. For example, Gram-positive bacteria have a high density of negative charges on the cell membrane, due to the high content of phosphate and hydroxyl groups, thus they are more vulnerable to cationic PSs.^60,61^ Taking this into account, the following classification of organic PSs have been introduced:

(1) Cationic PSs based on: phenothiazines,^62,63^ BODIPYs,^64,65^ phthalocyanines,^66^ porphyrins,^67^ or porphycenes.^68^

(2) Anionic PSs: xanthenes,^69–71^ and tricarbocyanine dyes.^72^

(3) Neutral PSs based on: chlorin,^73,74^ temoporfins^75,76^ or curcumin.^77,78^

The term Photodynamic Antimicrobial Chemotherapy (PACT) was introduced for the first time in 1960s. For many years, PACT was treated as a subgroup of anticancer Photodynamic Therapy (PDT) named Antimicrobial Photodynamic Therapy (aPDT), due to their similar mechanism of action. Nowadays, both terms: PACT and aPDT are commonly used in the literature. PACT employs photosensitizers to induce phototoxic effects in microorganisms. Inactivation of microbes using photosensitizers has been reported for various Gram-positive (*e.g. Staphylococcus aureus*, *Enterococcus faecalis*, *Bacillus cereus*) and Gram-negative bacteria (*e.g. Escherichia coli*, *Pseudomonas aeruginosa*),^79^ viruses (*e.g. Vesicular stomatitis*),^80,81^ and fungi (*e.g. Candida albicans*).^82^

The detailed mechanism of photosensitizers' antimicrobial action has been under debate for many years.^83^ Recently, Baptista *et al.*, proposed a classification of light-activated processes into (i) photosensitized oxidation and (ii) oxygen-independent photosensitization.^84^ The first one is based on the action of ROS that is generally multi-target and very efficient. As described above, reactive oxygen species are formed either *via* electron transfer (named as Type I aPDT) or by energy transfer (named as Type II aPDT).^85,86^ ROS interact with cell wall increasing the ion permeability, cause oxidation of proteins and structural changes in nucleic acids.^87–90^ Antioxidant enzymes can effectively protect microbes against some types of ROS, but not against ^1^O_2_. Within Type I, H_2_O_2_, O_2_˙^−^ and ˙OH are produced and the last is considered as the most reactive one in this group.^91^ Since ROS affects microbes in a multi-target mechanism, PACT can be advantageous for dealing with multi-drug-resistant microbes.^83,87^

The advantage of photoantymicrobials over biocides is that they are majorly nontoxic molecules that can kill microbes at lower conentrations than biocides and covers a whole spectrum of bacteria, viruses, fungi and protozoa.^92^ Photoactive compounds used in PACT should also prioritize physical properties as solubility and aggregation. Limiting the aggregation effect can reduce the dose of photosensitizer needed for the therapeutic effect.^93^

## Antimicrobial coatings based on organic photosensitizers

Similarly to the inactivation of microorganisms with non-immobilized photosensitizers, the action of light-activated antimicrobial coatings is based on the production of reactive oxygen species. Though, in this case, ROS photogeneration needs to be considered as a heterogeneous process, and additional parameters, *e.g.* transport of reactants to/within the layer, need to be taken into account.^94^

Till now, inorganic photoactive species have been widely used in the light-activated antimicrobial coatings. Next to titanium dioxide, which has been investigated for many years,^95–97^ zinc oxide has gained more interest in the last years.^3,98^ The main limitation of inorganic-based antimicrobial coatings is their absorbance located mainly in the UV region and their toxicity, which is still under investigation.^96,99^ Some of the photoactive coatings have already been approved for commercial use and introduced to the market, *e.g.* by PhotoACTIVE®,^100^ Envision^SQ^,^101^ TitanoClean™,^102^ and Pilkington.^103^

One of the first works discussing the possibility of the use of organic photosensitizers in the light-activated antimicrobial coatings was a series of papers of Wilson *et al.*, in which cellulose acetate was used as a matrix for Toluidine Blue O or Rose Bengal immobilization.^104–106^ So far, many papers reporting different strategies for the deposition of antimicrobial coatings based on organic PSs have been published.

Formation of organic layers, also photoactive ones, is possible using a variety of techniques that differ in the quality and properties of the resulting layer, ease of operation, control over the deposition process, or availability and costs of the equipment. For the aim of this review, the photosensitizers' immobilization techniques can be divided into two main groups resulting in (i) covalent or (ii) non-covalent interactions between PS and solid support.

A wide range of surface grafting techniques based on chemical, photochemical, electrochemical, or thermal processes can be used for the covalent attachment of organic molecules to solid surfaces. Within this group, Self-Assembled Monolayers (SAM) have been the most widely explored for many years. SAMs are formed thanks to the specific interactions between surface-anchoring groups present in organic molecules and a given type of surface, *e.g.* thiols – gold, alkoxysilanes – SiO_2,_ or indium tin oxide (ITO). The self-assembly process is spontaneous and the resulting layer usually possesses a well-defined and well-organized structure.^107,108^ The electrochemical grafting process, on the other hand, allows for the modification of (semi)conductive surfaces only. In most cases, the electrografting process is specific for the given type of substrate, *e.g.* oxidative electrografting of carboxylates occurring only on carbon surface.^109^ The most versatile electrografting technique, in terms of surface type and organic molecules, is an electrochemical reduction of diazonium salts^110^ that was reported for the first time in 1992 by Pinson *et al.*^111^ It has to be noted that thanks to so-called post-functionalization techniques, the chemical structure and thus properties of grafted organic layers can be further optimized.^112^ In the case of functionalization of polymers, two approaches can be distinguished: either functionalized monomers are directly polymerized or the so-called reactive polymer precursor and a consecutive postpolymerization modification are used.^113^

The second group of techniques, *i.e.* resulting in non-covalent deposition of organic layers is much broader and in most cases doesn't require introduction of any specific functional groups. The best example is physical adsorption of *e.g.* dyes, that is governed by van der Waals forces or hydrogen bonding.^114^ Here, we will discuss only a few examples of techniques that are the most frequently used for the preparation of the light-activated antimicrobial coatings.

In the solution-based techniques a substrate is uniformly coated with a solution of organic compound that after drying yields film. Those methods are very common both on the laboratory- and on the industrial scale, due to rather low-costs and ease of operation. Spin coating, dip coating, drop casting, and spray coating are examples of solution processing methods. They differ in the costs, speed of coating, the complexity of the process, uniformity of resulting film, *etc.*^115^ For example, the spin coating yields films with high uniformity, only small substrates can be coated and a straightforward scalability is not possible. On the other hand, in the spray coating large substrates can be covered quite quickly and the process is scalable, but the resulting layer has low uniformity and the costs are higher. In all the above-mentioned techniques the morphology, thickness, and uniformity of the film can be controlled by optimizing process parameters.^116–118^

Another technique in the non-covalent deposition group is electrochemical polymerization, which is used mostly for the formation of conducting polymer films.^119^ In this case, the functional group that undergoes electropolymerization needs to be present in the monomer's structure and the surface material needs to be conductive and not easily-oxidized, *e.g.* indium-tin-oxide on glass (ITO) or platinum plate (Pt) can be used. Finally, a non-covalent immobilization of PS in a polymer matrix may be achieved with methods well-known for enzymes^120^ or nanoparticles^121^ immobilization, *e.g.* encapsulation or entrapment.

Below, the selected examples of light-activated coatings based on organic photosensitizers are discussed. Table 1 summarizes the examples and gives additional ones, providing a reader with all necessary information about the type of the immobilized molecule, selected immobilization strategy, pathogen type or strain with the reported inactivation efficiency, *etc.*

**Table 1:** Organic light-activated antimicrobial coatings

No.	Photoactive molecule	Immobilization strategy	Antibacterial effect	Coating stability	Irradiation parameters	Ref.
1	Methylene blue (MB)	Immobilization in polystyrene	*S. aureus*: 1.5 log reduction. *E. coli*: 1 log reduction	Leakage under illumination was higher than that in the dark, resulting in 0.48 μM MB	0.5–3 h, white light, 400–700 nm, 1–3 mW cm^2^, light intensity (1.8–5.4 J cm^−2^)	130
2		Dispersed in polymer resins	*S. aureus, E. coli*: full bacterial growth; little growth; no growth – described	Not reported	4 min, 2 min, 1 min, 0.5 min, 615–645 nm, 48 mW cm^−2^	122
3		Covalently bound to a silicone surface	*E. coli:* 1.3 log reduction (energy dose of 42 J cm^−2^)	Not reported	21 min, 660 nm, 32.5 mW cm^−2^	123
4		Ultrasonic spraying, host–guest interaction between β-cyclodextrin and MB	*Methicillin-resistant S. aureus*: 99% reduction	Release of MB was observed	10 min, 660 nm laser (30 J cm^−2^)	179
5	Toluidine blue O (TBO)	Covalently bound to a silicone surface	*E. coli*: 2 log reduction (energy dose of 42 J cm^−2^)	Not reported	4 min, 634 nm, 190 mW cm^−2^	123
6		Deposited by absorption on the surfaces of the silicone and polyurethane polymers	*S. aureus*: >4 log reduction after 3 min (silicone), >4 log reduction after 1 min (polyurethane). *E. coli*: >4 log reduction after 2 min (polyurethane), 1.5 log reduction after 3 min (silicone)	When immersed in water or methanol, PS is not released into solution	1–3 min, 634 nm, 1.0 W laser	124
7		Immobilized in polymer matrix	*C. albicans*: Reduction >90%	Resisting dissolution when immersed in artificial saliva	3 h, 635 nm, 100 mW cm^−2^	125
8		Covalent linkage	*S. aureus*: 5–6 log reduction. *E. coli*: 5 log reduction	High charge density renders colloidal stability of the fibers	15 min, 630 nm red LED lamp and 300–800 nm solar simulator, 250W m^−2^	184
9	Rose bengal (RB)	(1) Plasma treatment + acrylic acid binding to PDMS. (2) Chemical grafting of chitosan-RB	*E. coli*: not able to inhibit the growth; *S. aureus*: vitality 50% inhibition	Increase in the surface wettability of PDMS samples after surface functionalization	60 min, incandescent lamp, 120 W	129
10		Immobilization in polystyrene	*S. aureus*: 3 log reduction. *E. coli*: 2.5 log reduction. *P*-values for comparison with control series were 0.0029 for *S. aureus* and 0.0038 for *E. coli*	Leakage under illumination was higher than that in the dark, resulting in 0.26 μM RB	0.5–3 h, white light, 400–700 nm, 1–3 mW cm^2^, light intensity (1.8–5.4 J cm^−2^)	130
11		Immobilization in polymeric matrix (PMMA, PC)	*S. aureus*: 99.998% reduction	Upon addition of RB, the porous structure was preserved only in the case of PS, whereas the RB-containing PC and PMMA had smooth surface structures	0–60 min, white light, 1.2 mW cm^−2^	9
12		Cross-linking to polymer	*E. coli*: 99 ± 2% reduction in dark, 28-fold increase upon irradiation; MRSA: >99 ± 1% reduction in dark, 3-fold increase upon irradiation; SARS-CoV-2: 90% reduction	Not reported	0.5-2h; 530 nm, 39 mW cm^−2^	127
13		Immobilized in Amberlite® by ion-exchange with chloride ions	*S. aureus*: 5.5–7 log reduction. *E. faecalis*: 8 log reduction *E. coli*: 5.5 log reduction *P. aeruginosa*: 8 log reduction *C. albicans*: 1.5–3.0 log reduction (dark effect)	Combination with commercial supports like cationic exchange resins enhances effectiveness	515 nm, total light dose of 100 J cm^−2^	128
14	MB,RB and TBO	PSs mixed with poly(vinylidene fluoride)	*E. coli*: 5 log reduction (24 h) *S. aureus*: 5 log reduction (6 h)	Soaking the PS surfaces in PBS for 1 week – stable in the surface and their leakage to the solution is negligible	6–24 h, white light, 1.46 mW cm^−2^	131
15	RB and TBO	Cellulose acetate modified with PSs	*S. aureus:* 78.9–99.8% inhibition	Not reported	6 h, fluorescent lamp (∼3700 lux), 28 W	106
16	Thionine	Grafting	*S. aureus*: 99.985% (∼3.82 log) reduction. *E. coli*: 99.99% (4 log) reduction	Presence of disperse dyes increases photostability; humidity negatively impacts stability	60 min, 400–700 nm noncoherent light, 65 ± 5 mW cm^2^; xenon lamp 500 W equipped with a long-pass filter (*λ* ≥ 420 nm)	132
17	Eosin Y	Immobilized during photoinduced crosslinking of a PEG–diacrylate monomer	*E. coli*: 4 log unit reduction. *S. aureus*: 4 log unit reduction	Not reported	6 h, visible light (400–650 nm)	133
18	Erythrosine B	Solvent casting	*S. aureus*: 5.4 log unit reduction. *E. coli*: undetectable *Salmonella*: Undetectable	Not reported; effectiveness decreased after multiple uses due to bacterial accumulation	10–50 min; LED light; 400–800 nm	134
19	Anthraquinone-triazine	Surface modification of cotton fabrics (covalently)	*E. coli*: 99.9% inhibition	The thermal decomposition occurred mainly in a narrow temperature range of 310–370 °C	2 days, 400–800 nm, 1380 lm, 110–130V, 400 mA	137
20	*Meso*-tetraphenylporphyrin (TPP)	Cross-linking	*S. aureus*: >99.9% reduction	Photostability under the tested conditions	30 min, 360–600 nm, 50 mW cm^−2^	138
21	Zn-porphyrin	Covalently attach	*Infuenza A virus*: Inactivated 99.99%	After exposure to high-intensity white light for 4 days and then subjected to a 1000 minute quantification experiment -similar levels of ^1^O_2_ production	4 h, visible light, 90 W	140
22	Zinc Tetra(4 *N*-methylpyridyl)-porphyrine (ZnTMPyP^4+^)	Spray-coating or dip-coating	*E. coli*: 99.86% inactivation *S. aureus*: 99.9999% inactivation SARS-CoV-2: 99.9998% inactivation	Not reported	1 h, 400–700 nm, *S. aureus* – 65 ± 5 mW cm^−2^, *E.coli* 80 ± 5 mW cm^−2^	141
23	ZnTMPyP^4+^, MB and RB	Spray coating	*S. aureus*: 97–99.999% inactivation HCoV-229E: 99.999% inactivation	Layers showed the same level of activity even after exposure for 4 weeks to indoor ambient lighting	1 h , 400–700 nm, *S. aureus* – 65 ± 5 mW cm^−2^, HCoV-229E – 80 mW cm^−2^	142
24	Pd(ii)-porphyrin	Electrochemical polymerization	*E. coli*: 3 log reduction *C. albicans*: 2.5 log reduction	Mechanically stable polymeric films	30 min, 60 min, 350–800 nm, 90 mW cm^−2^	143
25	Mn(iii) *meso*-tetra(4-sulfonatophenyl) porphine chloride (MnTPPS)	Electrostatic interaction – solvent evaporation	*E. coli*: 83% inhibition	Not reported	60 min, halogen bulb, 100 W	144
26	Hematoporphyrin	Covalent binding to stainless steel surface *via* esterification reaction	*E.coli*: decreased from 10^6^ CFU mL^−1^ to 10^4^ CFU mL^−1^. *S. aureus*: Decreased from 10^5^ CFU mL^−1^ to 10^0^ CFU mL^−1^	Not reported	2h, *λ*_max_ = 520 nm GLED, <400 nm cutoff filter, 3.5 mW cm^−2^	139
27	*Para*-aminophenylporphyrin derivatives	Grafting	*E.coli*, *S. aureus*: Inhibition are 37% for anionic cotton, 93.7% for neutral cotton, and 100% for cationic cotton	At much higher temperatures, grafted cotton samples showed multistep weight loss due to decomposition of photosensitizers, removal of linker groups, or degradation of polymeric material or backbone itself	24 h, 400–800 nm, 0.16 mW cm^−2^	146
28	*Meso*-arylporphyrin	Covalent linking to cellulose fabric *via* click-reaction	*E. coli*: 5.31 log CFU reduction. *S. aureus*: 5.3 log CFU reduction	Not reported	24 h, white light, 1000 lux	147
29	Protoporphyrin (PpIX)	Electrospinning	*E. coli*: 86.6% inactivation. *S.aureus*: 99.8% inactivation	Thermal stability of PpIX/CA microfibers in comparison to that of CA was not significantly changed: Both had a significant onset of decomposition at ∼300 °C	30 min, 420 nm, 65 ± 5 mW cm^−2^	148
30		*‘*One-pot, two-step’ reactions	*E. coli S. aureus* (The growth of the microorganisms was examined visually)	Not reported	Four 150 W tungsten bulbs, totalling 1.7 mW cm^−2^	149
31		Grafting	*E. coli*: 68.33–99.999% reduction, *S. aureus*: 98.50% reduction	Decreased efficacy after multiple uses due to photobleaching and bacterial accumulation	30 min, Xenon lamp (*λ* ≥ 420 nm)	150
32	Carboxyporphyrins	Grafting	*E. coli* and *S. aureus*: GP ≥ 0.18 photoinhibition	No change in UV-spectrum upon 48 h continuous illumination	24 h, four 150 W tungsten bulbs, 1.7 mW cm^−2^	151
33	Biscarbazol-triphenylamine end-capped dendrimeric zinc(ii) porphyrin	Electrochemical polymerization	*S. aureus*: >99.9998% reduction. *E. coli*: 99.4% reduction	Photostability observed with reusability for at least three treatments	15, 30, 60 min, visible light (455–800 nm), 0.5 mW cm^−2^	145
34	Tetra-substituted diazirine porphyrin cross-linked to polyethylene terephthalate	Cross-linking	*S. aureus*: 97.5% inhibition	Covalent cross-linking of porphyrin to PET provides long-term stability; stable antimicrobial properties	6 h, white LED light 75 W, 1800 lx, 59.37 J cm^−2^	152
35	Porphyrinic metal–organic frameworks (PCN-224) grown on Ti_3_C_2_ nanosheets	Magnetron sputtering	*E. coli*: 99.9999% inactivation *S. aureus*: 99.995% inactivation	Not reported	30 min, Xenon lamp, 420 nm filter, 500 W, 31.45 W cm^−2^	185
36	Ethynylphenyl porphyrin	Covalent attachment to cellulosic surface	*E. coli*: 1-2 log reduction. *M. smegmatis*: 3–4 log reduction. *S. aureus*: 5–6 log reduction	Minor degradation around 210 °C (weight loss of 20%) and major decomposition above 320 °C	30 min, 400–700 nm, white light 60 mW cm^−2^	186
37	5,10,15,20-Tetrakis(pentafluorophenyl)porphyrin	Polymerization	*E. coli*: 2.3 log reduction. *S. aureus*: 3.6 log reduction	After 75 minutes of irradiation, no significant loss of efficiency	75 min, white light, 156 mW cm^−2^	187
38	Tetrakis(4-carboxyphenyl)porphyrin (TCPP)	Surface-initiated polymerization and cross-linking	*E. coli*: 64% reduction	Minimal leaching of TCPP from the surface of SiO_2_ beads was observed after 7 days	24 h, 470 nm, LED, 2000 μW cm^−2^	153
39	Cationic zinc phthalocyanines	Electrostatic interactions	*E. coli*: 8 log reduction. *S. aureus*: 6 log reduction. *C. albicans*: 6.5 log reduction	Decomposition starts above 300 °C (Pristine cellulose crystals start to decompose above 200 °C)	620–645, red light irradiation, 18 mW cm^−2^	188
40	Zinc(ii) phthalocyanine tetrasulfonic acid, ZnPcS	Covalent attachment by reactive dyeing	*E. coli*: >2 log inhibition	Still stable after 9 months of use	30 min, visible light, halogen lamp, type no. CY-118A, 500 W 230 V, 50 Hz	189
41	Pyridine substituted phthalocyanine zinc complex	Dye-impregnated cellulose material	*C. albican* – 99.996% inhibition *S. aureus* – 99.998% inhibition *E. faecalis* – 99.998% inhibition	Not reported	30 min, 60 min, LED/fluorescent lamp, 4000 lux, 270 lux	190
42	Phenoxy-substituted phthalocyanine zinc (PPcZn)	Incorporation in cellulose acetate	Bacteriophage Qβ: 1.3 log reduction	The layer is still functional after 6 months of exposure to daylight	4 h, visible light, 1000 lux	182
43	Zinc tetracarboxy-phthalocyanine	Double-grafted: Solvent evaporation, dip-coating	*E. coli*: 99.99% reduction. *S. aureus*: 99.99% reduction	Not reported	30 min, LED, 680 nm	191
44		Grafting	*E. coli*: 99.99% reduction. *S. Aureus*: 99.99%reduction	Double-grafted fiber retained 99.75% of antibacterial efficacy after ten washing	10 min, 680 nm, 15 J cm^−2^	157
45	Mono-substituted β-carboxy zinc phthalocyanine	Grafting	*E. coli*: 99%, *S. aureus*: 98%	Coating was decomposing with time, but at much lower rate, comparing to photosensitizer in solution	10 min, 660–740 nm, 150 mW cm^−2^	158
46	Pyridine zinc phthalocyanine	Grafting	*A. baylyi*: 3.4 log reduction. *E. coli*: 2.7 log reduction	No leaching in water unless pH < 2; effective at 0.008 mg cm^−2^ loading	1 h, white light, 485–750 nm, 18 mW cm^−2^	156
47	Poly(3,4-ethylenedioxythiophene) zinc phthalocyanine (ZnPc-PEDOT)	Electrochemical polymerization	ZnPc-PEDOT:*S. aureus*: 99.98% reduction; *E. coli*: 99.98% reduction, CuPc-PEDOT:*S. aureus*: 90% reduction, *E. coli:* 95% reduction	Negligible photolysis of PS observed	ZnPc-PEDOT: 30 min (*S. aureus*) and 90 min (*E. coli*), visible light 108–162 J cm^−2^ CuPc-PEDOT: 60 min (*S. aureus*) and 90 min (*E. coli*), visible light 108–162 J cm^−2^	162
48	Silicon phthalocyanine derivative (AGA405)	Drop-casting	*E. coli*: 50% biofilm mass reduction	Not reported	30 min, near-infrared light (18 J cm^−2^); 30 min incubation	160
49	Bis-amino Si-phthalocyanine	One step sol–gel process	*P. gingivalis*: 99.99% reduction	No photobleaching in experimental conditions	15 min, CW diode laser 669 nm, 270–315 J cm^−2^	159
50	Tetratert-butyl-substituted silicon phthalocyanine dihydroxide	Adsorption	*S. aureus* 99.9% inactivation, *E. coli* unaffected	Not reported	1–3 h, polychromatic light (10 mW cm^−2^) with 610 nm cut-off filter; (108 J cm^−2^)	161
51	Chlorophyllin	Grafted to cotton fabric (Chl-fabric) or embedded in electrospinned polyacrylonitrile nanofibers (Chl-NF)	Chl-fabric: *E. faecium*: 99.998% reduction. *S.Aureus*: 99.994% reduction. *F. calcivirus*: 99.8% reduction. Chl-NF: *E. faecium*: 99.9999% reduction. *S.Aureus*: 99.9999% reduction. F. calcivirus: 99.8% reduction. K. pneumoniae: 99.9999% reduction after addition of MoS_2_	Chl-fabric: Photostability for short-term use. Chl-NF: Higher photosensitizer loading, better inactivation	30–60 min, 400–700 nm, 80 ± 5 mW cm^−2^ for lower intensities LED light 3 ± 1 mW cm^−2^ and LumaCare PDT light 30 ± 5 mW cm^−2^	164
52	8-Acetoxymethyl-2,6-dibromo-1,3,5,7-tetramethyl pyrromethene fluoroborate (Br_2_B-OAc)	Spin coating	*S*. *aureus*: >5 log inactivation. *E. coli*: 3.5 log inactivation	Stable; 7% reduction in fluorescence intensity after 1 month in water and ambient light	30 min, 480−550 nm, 1.0 mW cm^−2^	166
53	Boron-dipyrromethane) (BODIPY) derivative	Covalent attachment to PDMS	*S. aureus* – 99.9% reduction	Not reported	5 h, white light, 4000 lux	167
54	Paprika spice	Photopolymerization	100% inhibition of adhered *E. coli* and *S. aureus* after 2 h and 6 h, resepctively	Thermal stability up to 300 °C	Visible light, total intensity of 170 μmol m^−2^s^−1^	192
55	Quercetin	Spin coating	*S. aureus*: 99% reduction	Very good adhesive properties to the stainless steel substrates and a high thermal stability up to 375 °C	2 h and 6 h, 365 nm, Xe lamp, 70 mW cm^−2^	193
56	Curcumin	Photopolymerization	*E. coli*: 95% inactivation. *S. aureus*: 99% inactivation	Good adherence properties on an inox substrate and a high thermal stability to 375 °C	48 h, 4 lamps, 170 μmolm^−2^s	194
57		Atom-transfer radical polymerization	*E. coli*: ∼2 log reduction	Surface morphology does not change after immersion in water	15 min, white-light, 42 mW cm^−2^	168
58	Triphenylamine quinolinium hexafluorophosphate (TPAQ-PF6)	Covalently grafted	*S. aureus*: 91.0% reduction. *P. aeruginosa*: 97.0% reduction	The material retained its antibacterial efficacy even after multiple washing cycles	60 min, white light, 40 mW cm^−2^	195
59	Boron-functionalized polyethyleneimine (PEI-BF_2_)	LbL assembly	*E. coli*: 99% reduction. *S. aureus*: 99.9% reduction	No change in thickness was observed over 3–4 days, indicating the photostability, stable in the pH range of 4–10	12 h, visible light irradiation, 12 V, 36 W	196
60	Acridine	Spin-coating, spray-coating, drop-coating	*E. coli*: 46.0% inhibition. *S. aureus*: 61.2% inhibition. *P. aeruginosa*: 75.4% inhibition. 99.3% inhibition of all of the above after 1 month	The contact angle of the surface did not change considerably after being treated with acid, alkali, salt, and other liquids for 48 h, 2 h of UV irradiation, 2 L running water attack, and 2 h of ultrasonic shaking	60 min, visible light	197

## Phenothiazines and xanthenes

Some of the most widely-explored photosensitizers, either in solution or in coatings, are methylene blue (MB), toluidine blue O (TBO), and rose bengal (RB) (Fig. 3). The first two are phenothiazine derivatives, *i.e.* cationic PSs, characterized by low toxicity and strongly absorbing in the range 500–750 nm. In one of works of Wainwright *et al.*, phenothiazinium-based coatings with methylene blue were prepared by the solvent evaporation method. The resulting films showed antibacterial activity against *S. epidermidis* and *E. coli* under illumination.^122^ When bound covalently to a silicone surface, MB also showed good bactericidal properties. In addition, when compared to TBO, it yielded higher efficiency of inactivation of methicillin-resistant *Staphylococcus aureus* (MRSA).^123^ However, TBO deposited on polyurethane showed better bactericidal properties than on silicone and amounted to >4 log after 2 min exposure and 1.5 log after 3 min exposure respectively. Similarly, for MRSA the detection limit (reduction >4 log) was reached after 3 min when silicone was employed and after 1 min when polyurethane was used. Compared to inorganic antimicrobial coatings, *e.g.* with titanium dioxide, it takes 4–24 hours to kill MRSA bacteria up to this level.^124^ It was stated by the authors that even a short-time application of TBO-containing mucoadhesive patches should make it possible to treat freshly acquired oral and pharyngeal candidiasis.^125^

Rose bengal is a water-soluble anionic photosensitizer known for a high singlet oxygen quantum yield (*Ф* = 0.79). RB immobilized in PC (polycarbonate) and PMMA (poly(methyl methacrylate)) exhibited high antibacterial activity. The amount of *S. aureus* dropped by 3–3.5 orders of magnitude after 0.5 h of illumination and an additional *ca.* tenfold decrease was observed after 1 h of treatment.^126^

**Fig. 4:**
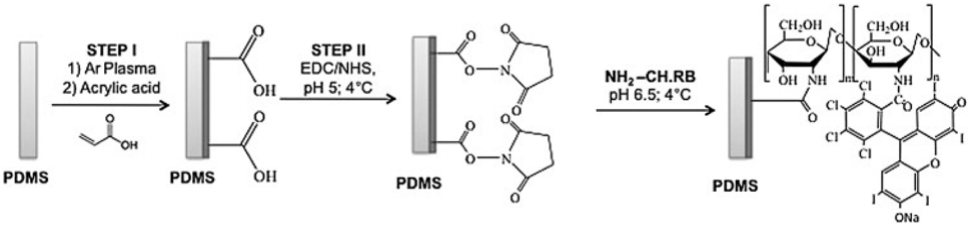
Schematic illustration of the different steps for PDMS surface modification with CH. RB. Reprinted from ref. 129 Copyright (2013), with permission from Elsevier.

In the work of Wright *et al.*, rose bengal lactone was photolinked onto a siloxane copolymer. The produced textile showed both photo-activated and contact-antimicrobial properties. The >98% inactivation was observed for *S. aureus* and *E. coli*, and was further increased in the presence of light – 3× and 28×, respectively. In addition, the removal of Severe acute respiratory syndrome coronavirus 2 (SARS-CoV-2) up to 90% was observed.^127^ Valkov *et al.* showed RB cross-linked to commercially available cationic polystyrene formed antimicrobial light-activated coating, which is efficient in *E. coli*, *Enterococcus faecalis*, *S. aureus*, and yeast *Candida albicans* inactivation using green light (5–8 log).^128^ Finally, RB layers combined with chitosan on PDMS (poly(dimethylsiloxane)) (Fig. 4) resulted in an approximately 50% inhibition of *E. coli*.^129^

Since most organic PS possess rather narrow absorbance bands, sunlight or indoor lighting is not fully used to produce ROS. One of the ways to overcome this problem and increase aPDT under white light is the formation of a coating containing various PSs having complementary absorption. In one of the works, MB and RB were immobilized by mixing solutions of the photosensitizers in chloroform with a polymer solution. This was followed by air evaporation of the solvent. The obtained polymer films showed significant antimicrobial properties, resulting in a 1.5–3 log reduction in *S. aureus* and *E. coli*.^130^ In another work, the combination of MB, RB, and TBO resulted in *E. coli* inactivation up to 4 log levels after 24 hours. However, a reduction of over 4 log in *S. aureus* was observed even after just 6 hours of irradiation. This demonstrates an inexpensive, straightforward, and contemporary approach to the preparation of antibacterial surfaces.^131^ In the work from Decraene *et al.*, *S. aureus* suspended in phosphate-buffered saline (PBS), saliva, or horse serum was sprayed onto cellulose acetate coatings containing TBO and RB, and survival of the organism on these surfaces was determined after 6 h of exposure to a household light source. Inactivation ranged from 78.9% (in horse serum) to 99.8% (in PBS) was reported.^106^

Thionine was grafted to the cotton fiber using cyanuric chloride (2,4,6-trichloro-1,3,5-triazine) as the coupling agent. Produced light-activated coating showed 99.985% (∼3.82 log unit reduction, *P* = 0.0021) effectiveness against *S. aureus*, and 99.99% (4 log unit reduction, *P* ≤ 0.0001) against *E. coli* and 99.99% inactivation against enveloped human coronavirus 229E.^132^ Thionine aggregation at high concentrations reduces its photosensitizing efficiency by hindering light absorption and energy transfer, leading to lower singlet oxygen generation. The presence of dispersed dyes can improve the photostability of the system by preventing the degradation of thionine under prolonged light exposure, thus maintaining its antimicrobial activity. However, humidity negatively affects the stability of photosensitizers, as moisture can cause hydrolytic degradation and interfere with the photosensitizer's electronic properties.^132^

The coating obtained by photoinduced cross-linking of a PEG–diacrylate monomer associated with the eosin Y dye showed antibacterial activity against *E. coli* and *S. aureus* under white light through ROS generation mechanisms and in the dark *via* antibacterial agent release from the coating.^133^ The solvent casting method was employed to produce a light-activated coating with erythrosine B acting as a photosensitizer. It was efficient in photodynamic inactivation of *S. aureus*, *E. coli*, and *Salmonella*. The authors suggest using this prototype for the development of photodynamic antibacterial and environmentally friendly active packaging material.^134^

## Anthraquinones

**Fig. 5:**
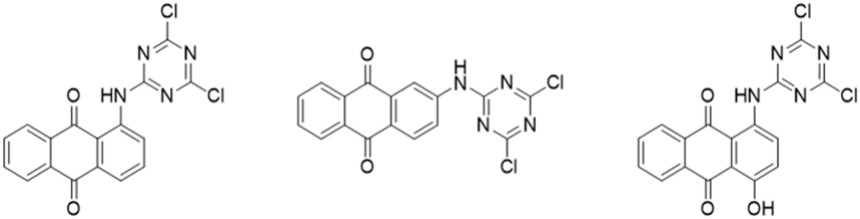
Anthraquinone-triazine structures for surface modification of cotton fabrics investigated in ref. 137.

Aminoanthraquinone dyes (ANQ), used for semi-permanent hair coloring and dyeing fabrics and plastics, show singlet oxygen yield equal to *ca.* 84%.^135^ Photochemical studies on natural anthraquinones have revealed that their triplet state can promote the formation of reactive species, such as the superoxide radical anion, and that they are also highly effective photosensitizers for singlet oxygen production.^136^ Three anthraquinone dyes (Fig. 5) covalently bonded to cotton fabric demonstrated effective antimicrobial properties under visible light exposure, achieving a 3 log inactivation efficiency against *E. coli*.^137^

## Porphyrins

**Fig. 6:**
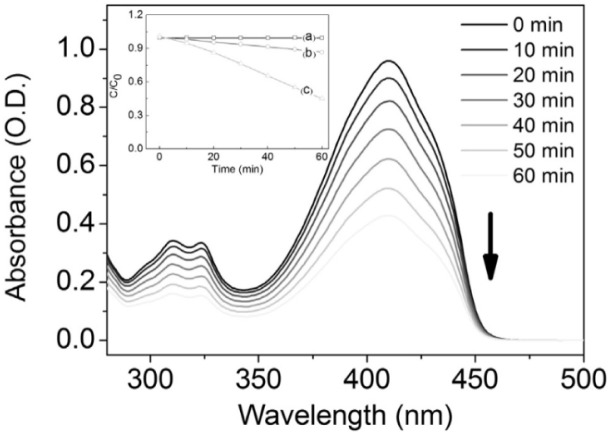
Set of UV-vis spectra of DPBF in ethanol in the presence of photofunctional stainless steel and under green light irradiation. Inset represents the ratio between the sajewing concentration and concentration of DPBF in time of the measurement: (a) DPBF with the PSS plate under dark conditions (b) DPBF only with the light, (c) DPBF with the PSS plate with the light. All measurements were performed with the same power of the light irradiation. Reprinted fromref. 139. Copyright (2017), with permission from Elsevier.

Porphyrin is a conjugated macrocycle made of 4 pyrrole rings with a high molar absorption coefficient (*ε*), excellent photostability, and biocompatibility with mammalian cells. In the work of Felgenträger *et al.*, *meso*-tetraphenylporphyrin was deposited on polyurethane through a spraying and the produced coating demonstrated remarkable efficacy in the photodynamic inactivation of *S. aureus* – more than 99% (>3 log-steps) yield within 30 minutes irradiation.^138^ In another work, hematoporphyrin (HP) was covalently bonded to the surface of 316L stainless steel through an esterification reaction. The antimicrobial effect of the PSS plate was tested with *S. aureus* and *E. coli*. The formed biofilm by *S. aureus* was effectively inactivated (99.999%). The biofilm formation by *S. aureus* was efficiently inhibited for 2 days under the condition of light irradiation. The confirmation of reactive oxygen generation was done by measuring the time-dependent UV-vis spectra of DPBF (1,3-diphenylisobenzofuran) – a chemical trap for singlet oxygen (Fig. 6).^139^

The introduction of a central metal atom to the porphyrin core strongly alters its properties – absorbance, photoactivity, or (photo)stability. Thus, metalloporphyrins are also widely investigated for aPDT processes. For example, zinc porphyrin was attached to the surface of a melt-blown non-woven textile filter material. The resulting material exhibited remarkable performance against the *Influenza A* virus, achieving a 99% effectiveness. Interestingly, when the sample was exposed to high-intensity white light for 4 days and then subjected to a 1000 minutes ^1^O_2_ quantification experiment, there was a similar level of ^1^O_2_ production, which confirms the stability of the system.^140^ In another work, zinc 5,10,15,20-tetrakis(4 *N*-methylpyridyl)porphyrin (ZnTMPyP^4+^) tetrachloride was employed in the fabrication of light-activated layers through two distinct coating methodologies: spray-coating and dip-coating with PA6. Produced coatings were found very effective in the reduction of *S. Aureus*, antibiotic-resistant *E. Coli*, and SARS-CoV-2.^141^

Three types of coating, composed of the commercially-available UV-photocrosslinkable polymer *N*-methyl-4(4′-formyl-styryl)pyridinium methosulfateacetal poly(vinyl alcohol) (SbQ-PVA) and one of three photosensitizers- (ZnTMPyP^4+^), methylene blue or rose bengal, showed clear bioinhibition of *S. aureus* and the human coronavirus strain HCoV-229E under visible light illumination with efficiency ranging from 97–99.999% and HCoV-229E inactivation from 92–99.999%, even after exposure for 4 weeks to indoor ambient room lighting, depending on the employed photosensitizer. This is proof of the long duration of action and stability of these porphyrin derivatives, which is a great advantage when used as self-disinfecting surfaces.^142^ These results are extremely promising, indicating that the porphyrins can be applied (in the form of a coating) as well as in fibrous furnishings found in homes, offices, temporary housing, and medical facilities.

Funes *et al.* reported, two porphyrins coatings (5,10,15,20-tetra(4-*N*,*N*-diphenyl aminophenyl)porphyrin (H_2_P-film) and its complex with Pd(ii) (PdP-film)) were created on optically transparent indium tin oxide (ITO) electrodes using the electrochemical polymerization. The resultant layers exhibited an approximate 3 log reduction in *E. coli* and a 2.5 log reduction in *C. albicans* cellular survival after 30 minutes of irradiation with visible light.^143^ Lower levels of pathogen inactivation (87% against *E. Coli*) were observed for micrometer-sized porous honeycomb thin films formed using hybrid complexes formed by electrostatic interaction between *meso*-tetra(4-sulfonatophenyl)porphine chloride Mn(iii) (acid form, {MnTPPS}) and dimethyldiocta-decylammonium bromide (DODMABr). The reduction of bacteria in the light was 83%, while the reduction in the dark for honeycomb films was only 5%.^144^ The electrochemical polymerization *via* oxidation of the carbazole groups was used to obtain biscarbazol-triphenylamine end-capped dendrimeric zinc porphyrin, which successfully eliminated *S. aureus* and *E. coli.*^145^

**Fig. 7:**
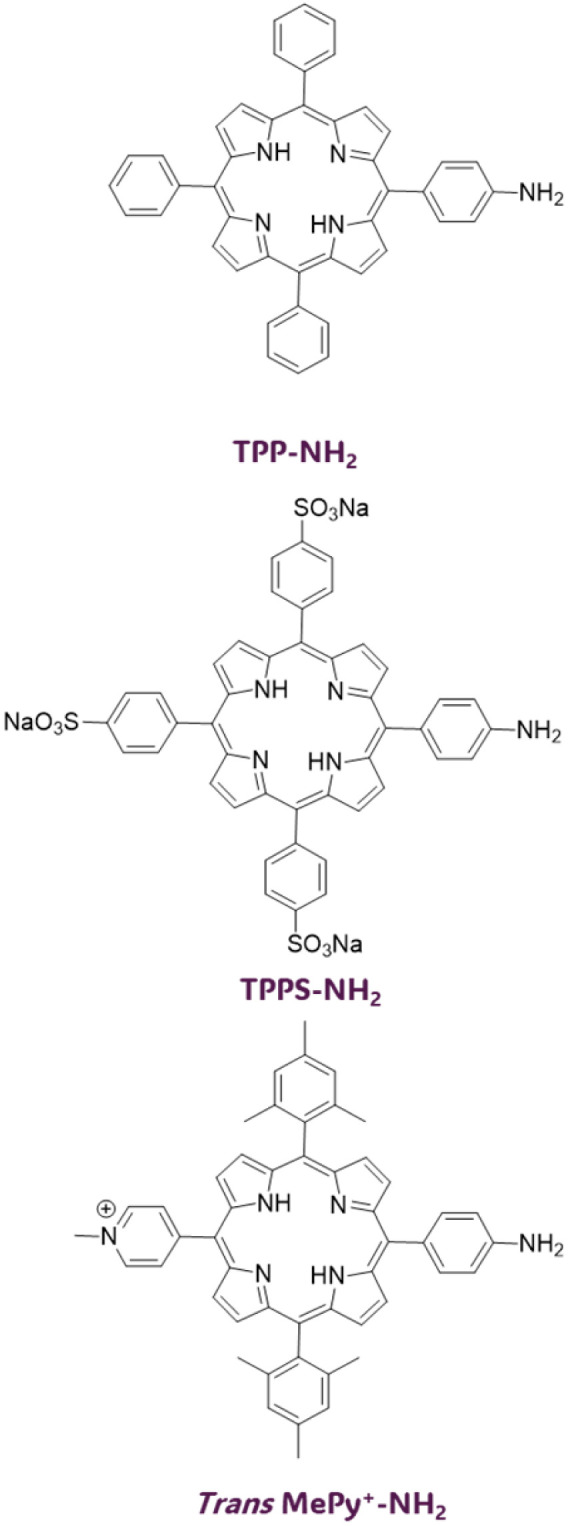
Chemical structure of neutral, anionic, and cationic amino porphyrins investigated in ref. 146. Adapted with permission from ref. 146. Copyright 2011, American Chemical Society.

**Fig. 8:**
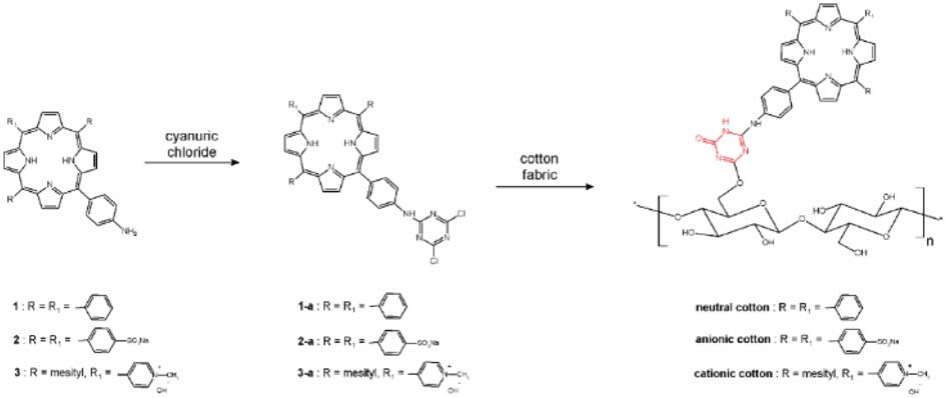
Synthetic route to photoantimicrobial cotton. Reprinted with permission from ref. 146. Copyright 2011, American Chemical Society.

In the work of Krausz *et al.*, anionic, neutral, and cationic amino porphyrins (Fig. 7) have been covalently grafted onto cotton fabric using 1,3,5-triazine derivative as the linker.^146^ The following modifications were implemented for the click–chemistry reaction (Fig. 8): porphyrins were reacted with cyanuric chloride, enabling the substitution of the first triazine chlorine atom with an amino group, yielding porphyrin-triazine derivatives. This was followed by the complete substitution of chlorine atoms using piperidine and sodium sulfonate. Finally, alkali-treated fabrics (cellulose) were introduced into the reaction mixture, and the grafting process was followed by washing cycles to remove any unreacted photosensitizer. The resulting coatings, with neutral and anionic porphyrin as photosensitizers, were effective in the inactivation of *S. aureus* and *E. coli.* Depending on the photosensitizer charge, different degrees of bacterial inhibition were obtained. Percentages of bacterial growth inhibition are 37% for anionic cotton, 93.7% for neutral cotton, and 100% for cationic cotton. The electric charge of photosensitizers directly influences photoinactivation efficacy, and these results confirm the presence of a structure–activity relationship in the photoinactivation of Gram-positive bacteria.^146^ The click-chemistry approach was also used for grafting *meso*-arylporphyrin to cotton fabric using cellulose azidation followed by acetylenic porphyrin. *Meso*-arylporphyrin-appended polymers inactivated Gram-negative and Gram-positive bacteria.^147^

**Fig. 9:**
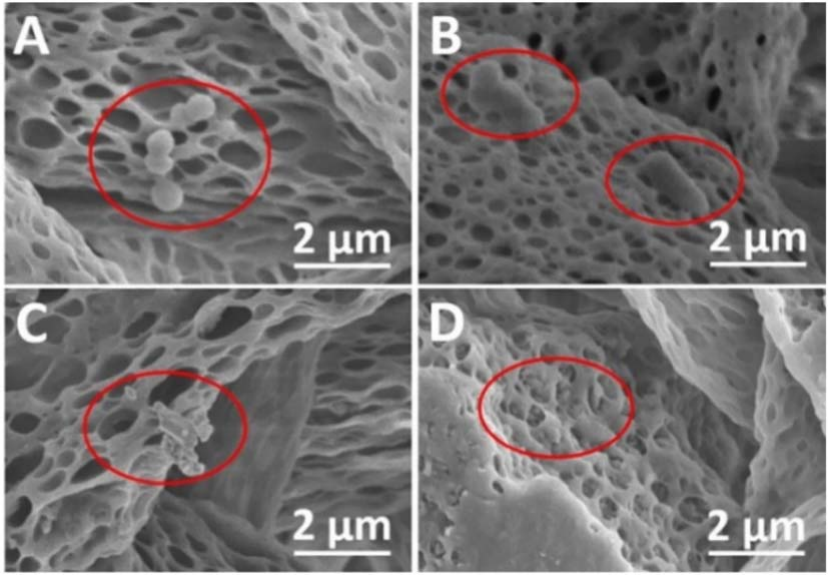
SEM images of *S. aureus* and *E. coli* on the PpIX/CA microfibers before (A and B, respectively) and after (C and D respectively) illumination. Reproduced with permission from ref. 148 Copyright 2021, Elsevier.

**Fig. 10:**
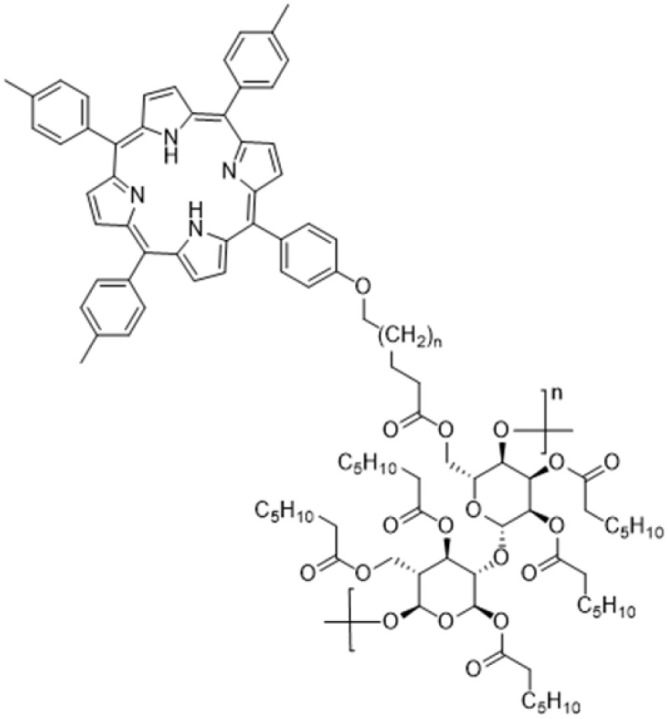
Structure of cellulose laurate ester modified with porphyrin investigated in (ref. 151).

Wang *et al.* showed that electrospun microfibers of cellulose diacetate (CA) with embedded protoporphyrin IX (PpIX) can effectively inactivate *S. aureus* and *E. coli* (99.8% and 86.6% photodynamic inactivation, respectively).^148^ The bacteria were investigated in detail using scanning electron microscopy (SEM). The obtained SEM images confirmed the irreversible oxidative damage due to ROS (Fig. 9).^148^ PpIX was also used as a photosensitizer in the work of Krouit *et al.*^149^ and the work of Dong *et al.*^150^ In both cases, the covalent immobilization of PS was obtained, either in a “one-pot, two-step” esterification approach or diamide spacer, respectively. “One-pot, two-step” grafting *via* esterification was also proved to be effective for immobilization of carboxyporphyrins (Fig. 10) to cellulose laurate esters. It was shown that by varying the length of the alkyl chains, the problem of steric hindrance can be overcome. The grafted carboxyporphyrins showed efficiency in inhibiting bacterial growth of *E. coli* and *S. aureus.*^151^

Cross-linked to polyethylene terephthalate tetra-substituted diazirine porphyrin formed an antimicrobial coating with bactericidal properties towards *S. Aureus* (1.76 log inactivation). The cross-linking was performed *via* a thermally triggered C–H insertion mechanism, activating the diazirine moieties to lose dinitrogen and form stable C–C bonds with the substrate, making the produced coating covalently attached and resistant to photobleaching.^152^

In the work of Hunter *et al.*, the functionalization of SiO_2_ with polymer brushes and crosslinking them with carboxylic acid-functionalized porphyrins (TCPP), was confirmed through various characterization techniques. The modified SiO_2_ beads exhibited enhanced singlet oxygen production under visible light, leading to effective antibacterial activity against *E. coli*.^153^

## Phthalocyanines

Another important class of organic photosensitizers is phthalocyanines (Pcs). Thanks to extended conjugation, phthalocyanines absorb in longer wavelengths than porphyrins, thus they are widely-investigated for application in PDT.^17^ The photophysical and photochemical properties of Pcs can be tuned by varying central metal atoms or outer substituents.^154,155^ Zinc phthalocyanine is one of the most studied Pc-based photosensitizers, due to its high quantum efficiency of singlet oxygen generation. In the work of George *et al.*, novel pyridine zinc phthalocyanine was synthesized and immobilized on filter paper *via* adhesion. The resulting material demonstrated *ca.* 3 log reduction in CFU against *E. coli* and *A. baylyi* ADP1 just after 1 h of 16 illumination with the white light of low intensity.^156^

**Fig. 11:**
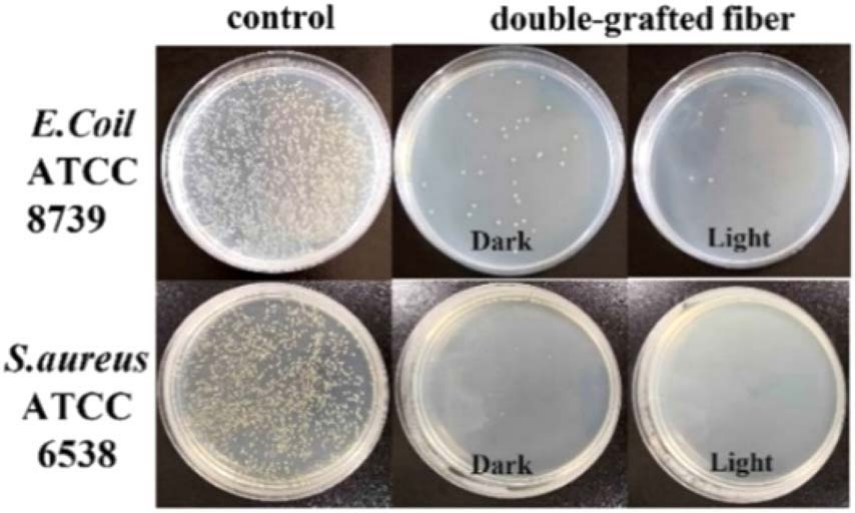
Antibacterial activity of the fiber materials using colony counting method. Reprinted with permission from ref. 157. Copyright 2022, American Chemical Society.

**Fig. 12:**
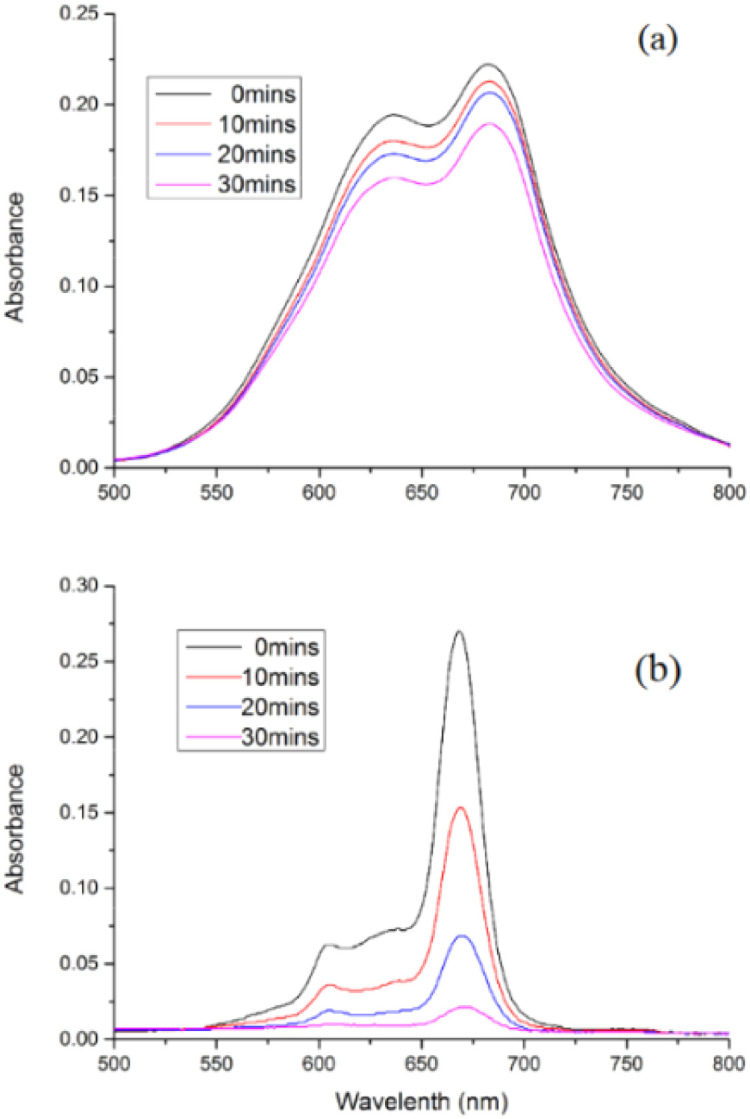
High photostability of CPZ-EPL-Fabric (a) as shown by the much slower photo-bleaching rate compared to CPZ in methanol (b). 150 mW cm^−2^ energy density. Reproduced with permission from ref. 158 Copyright 2017, Elsevier.

In the recent work of Lin *et al.*, zinc tetracarboxy-phthalocyanine was grafted to a fibrous PET that was followed by chitosan coating on the modified fiber. The resulting photoactive material was capable of significant biofilm inhibition with 3 log against *E. Coli* and *S. Aureus.* Importantly, under dark conditions, the double-grafted fibers also showed high efficiency (Fig. 11). The double-grafted fiber retained 99.75% of antibacterial efficacy after ten washing.^157^ In another study on zinc carboxy-phthalocyanin *e*, coating fabric was produced. In the first layer, ε-polylysine with positive charges significantly disrupts bacterial membrane, while the second layer contains ZnPc for aPDT action. This coating efficiently inactivated *E. Coli* and *S. Aureus* by 99% and 98%, respectively. Notably, the photostability of zinc carboxy-phthalocyanine is increased when immobilized, as shown by the photobleaching tests (Fig. 12).^158^

**Fig. 13:**
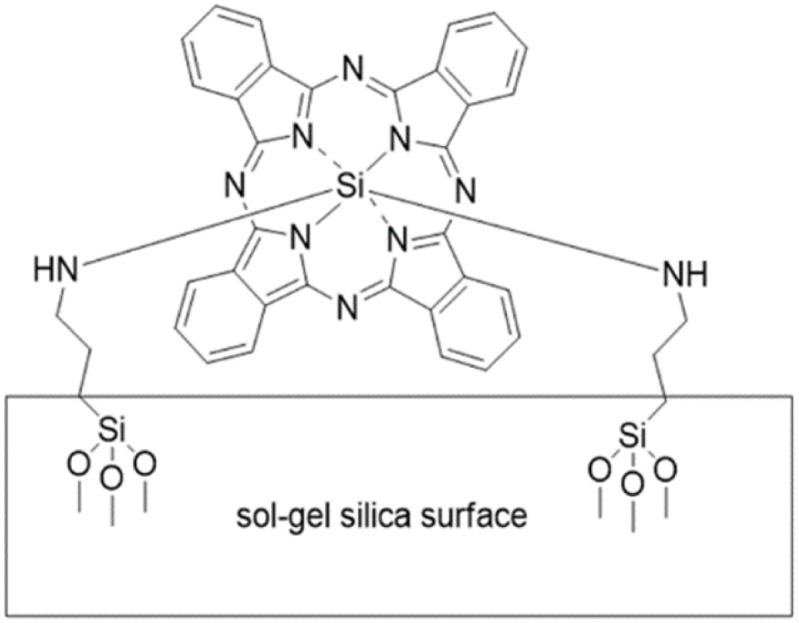
Bisamino Si-phthalocyanine incorporated by a sol–gel investigated in. (ref. 159) Adapted with permission from ref. 159. Copyright 2018 American Chemical Society.

In the work of Pushalkar, silicon phthalocyanine (SiPc) was covalently attached to a sol–gel silica surface (Fig. 13). The resulting system exhibited a significant yield of ^1^O_2_ production (2.3-fold higher quantities than for modification with chlorin e6). The biofilm inactivation (>5 log reduction) of *Porphyromonas gingivalis* was reported. The bacterial cultures were cultivated on hydroxyapatite discs (discs were selected to mimic the conditions of the teeth surface since hydroxyapatite is the primary mineral found in teeth), underscoring the potential of the developed device for the treatment of periodontitis.^159^

**Fig. 14:**
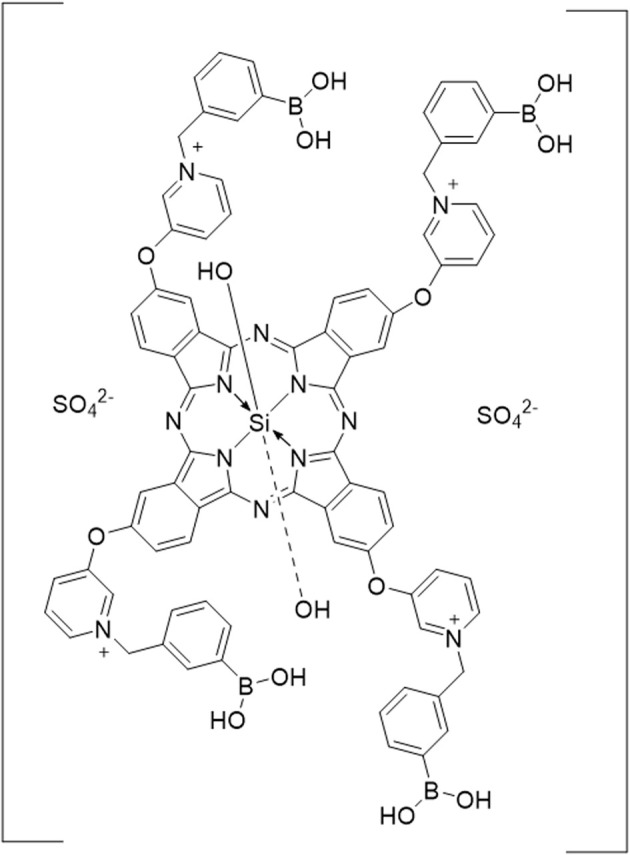
Silicon phthalocyanine derivative investigated in. (ref. 160) Adopted with permission from. (ref. 160) Copyright 2017, Wiley-VCH.

**Fig. 15:**
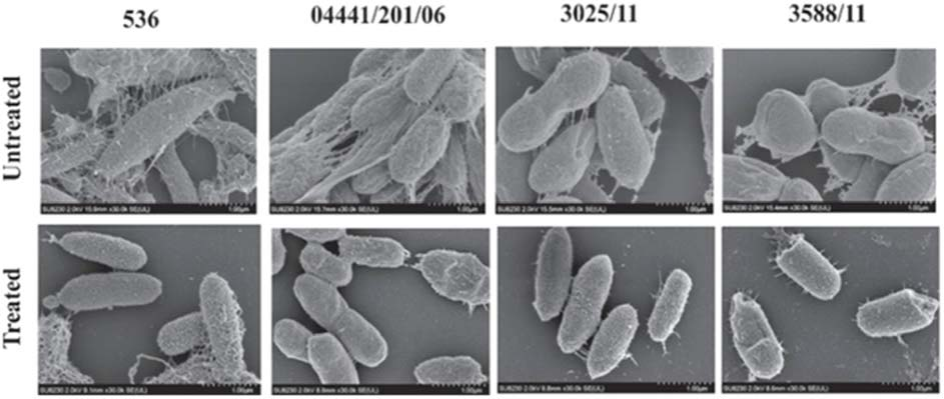
Representative scanning electron microscopy (SEM) images of treated and untreated biofilm samples. Reproduced with permission from ref. 160. Copyright 2017, Wiley-VCH.

Silicon phthalocyanine derivative (AGA405, Fig. 14) was linked to poly(vinyl alcohol) (PVA) *via* boronic acid and drop-casted. The produced PVA-AGA405 coating showed antibacterial activity, sufficiently inhibiting the growth of *E. coli.* SEM was applied for the visualization of bacterial cells and extracellular surfaces of biofilms (Fig. 15), proving the changes in the morphology of bacteria due to ROS.^160^

A straightforward adsorption of axially and peripherally substituted silicon phthalocyanines were used to modify LAPONITE^®^ nanodiscs. It was shown that SiPc loading strongly influences the photoactivity of the system. The resulting systems were effective towards *S. Aureus* and no effect towards *E. Coli* was observed. This was explained by interactions between the surface of the modified nanodiscs and the peptidoglycan layer of Gram-positive bacteria, while for the outer membrane of Gram-negative bacteria such interactions are hindered.^161^

Baigorria *et al.* reported electropolymerized ZnPc-PEDOT and CuPc-PEDOT coating having antimicrobial activity towards *S. Aureus* and *E. Coli,* It was shown that the incubation of surfaces in 0.1 M KI solution prior to photodynamic inactivation experiment improved the efficiency of phototherapy at least two times for both types of coatings, showing the synergizing effect of singlet oxygen and iodine species.^162^

## Chlorins

Chlorins as tetrapyrrole-based compounds stay closely related to porphyrins and phthalocyanines. Chlorins are highly effective photosensitizers, owing to their high singlet oxygen quantum yields (*e.g.* 89% for 2-chloro and 98% for 2,6-dichlorophenyl derivatives)^163^ In the recent work of Jiang *et al.*, chlorophyllin was grafted to cotton fabric or embedded in electrospinning polyacrylonitrile nanofibers. Modified cotton fabric and nanofibers effectively generated ROS and inhibited the growth of *E. faecium* and *S. aureus*. Slightly higher effectivness, *i.e.* 99.9999%, was observed for nanofibres.^164^

## Other

Apart from classical photosensitizer groups presented above, novel classes are being widely investigated. The main aim is to develop PS with boosted properties, *e.g.* absorption range, absorption coefficient, photostability, or efficiency of ROS production. Boron-dipyrromethanes (BODIPY), metal complexes, or perylenebisimides are widely explored.^165^ Though, most of the works on novel photosensitizers reported their efficiency in the solution phase, several works already reported their application in the light-activated antimicrobial coatings.

**Fig. 16:**
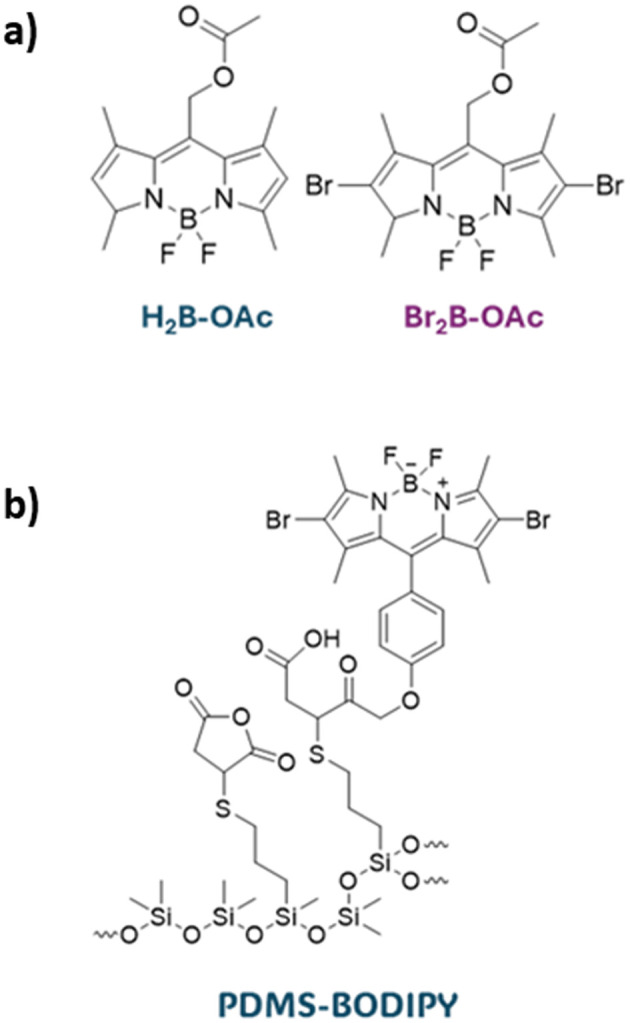
(a) BODIPY derivatives investigated in ref. 166 (b) PDMS-BODIPY investigated in ref. 167.

In the work of Martinez *et al.*, spin-coating was used for the deposition of 8-acetoxymethyl-2,6-dibromo-1,3,5,7-tetramethyl pyrromethene fluoroborate (Br_2_B-OAc) (Fig. 16a). The produced coating successfully inactivated *S. aureus* and *E. coli* in planktonic media for at least three cycles with short-time light exposure.^166^ BODIPY-derivative was covalently linked to a poly(dimethylsiloxane) material (Fig. 16b) to give material effective against *S. aureus* biofilm growth.^167^

The use of natural organic photosensitizers conduces to the formation of biocompatible layers with uniform antimicrobial properties. In the work of Santos *et al.*, an example of a layer in which cationic polymeric biocides (SPB) were combined with a natural photosensitizer – curcumin, was described. Studies using Gram(−) and Gram(+) bacteria did not show a significant difference in their antimicrobial activity in dark or light conditions, probably due to the small amount of PS present in the bacterial suspension, which did not produce enough reactive oxygen species (ROS) to have a lethal effect on microorganisms.^168^

## Hybrid coatings

Hybrid materials, formed by joining organic units with inorganic ones, are formed to obtain systems showing better properties than its individual counterparts. In the last years, such systems have attracted a great attention, since thanks to combining the valuable properties of their building blocks, they can be favourable for application in *e.g.* optics, electronics, energy storage, medicine. Similar approach can be undertaken in the case light-activated antimicrobial coatings, in which organic photosensitizers can be combined with photoactive inorganic or carbon nanomaterials (Table 2).

**Table 2:** Hybrid light-activated antimicrobial coatings

No.	Hybrid system	Immobilization strategy	Antibacterial effect	Irradiation parameters	Ref.
1	Crystal violet with cadmium-free quantum dots	Swell-encapsulation-shrink method, incorporation into polyurethane	*methicillin-resistant Staphylococcus aureus*: 99.98% reduction. *E. coli*: 99.96% reduction	18 h for *S. Aureus* and 4 h for *E. Coli*; broad-band visible illumination at 6000 lux	169
2	Crystal violet, ZnO nanoparticles	Two step dipping process, incorporation in acrylic latex	*E. coli*: 1.97–2.51 log reduction for CV-only (4 h). *S. aureus*: 1.16–2.01 log reduction for CV-only (3 h, 1.34 log higher for CV-ZNO)	2 h-6 h, white light, 512 lux	170
3	Phloxine B, layered silicate, polyurethane	Nanocomposite supported on polytetrafluoroethylene	*S. aureus*: 4 log reduction	120 s irradiation with green laser (532 nm, 100 mW)	171
4	Erythrosine B, layered silicate, polyurethane	Nanocomposite supported on polytetrafluoroethylene	*S. aureus*: Up to 10.000-fold reduction	10 min green laser 1.5 h green LED light	172
5	Methylene blue, crystal violet, Au nanoparticles	Silicone surface modification	*S. epidermidis*: ≥2.92 log reduction (3h). *S. cerevisiae*: 1.5 log reduction (3 h). MS2 Bacteriophage: 2.33 log reduction (4 h)	1 h – 5 days, fluorescent tube light, 8 W, 3500 lux	173
6	Protoporphyrin IX (PPIX-ED), Ag nanoparticles	(1) Bio-inspired cationic polymer bearing pendent catechols; (2) silver-loaded nanogel decorated with o-quinone groups; (3) amino modified protoporphyrin IX	*B. subtilis*: 14.0 mm of inhibition zone. *E. coli*: 17.4 mm of inhibition zone	24 h, 380 – 750 nm, 300 W	174
7	Poly(3,4-ethylenedioxythiophene)-fullerene C_60_ (PEDOT-fullerene C_60_)	Electrochemical polymerization	*S. aureus*: >99.9% inactivation	15, 30, or 60 min, visible light (3.1 mW cm^−2^, 5.6 J cm^−2^); different light doses compared to previous studies	175
8	Porphyrin-fullerene C_60_ dyad (TCP-C_60_)	Electrodeposited film	*S. aureus*: 4 log reduction. *E. coli*: 4 log reduction	30 min (*S. aureus*) 60 min (*E. coli*), 350–800 nm, 90 mW cm^−2^	176

Owusu *et al.* reported cadmium-free quantum dots and crystal violet conjugates immobilized *via* the swell-encapsulation method. The produced coating was efficient in the inactivation of *E. Coli* and *S. Aureus* with 99.96% and 99.98% reduction, respectively.^169^ In another work, crystal violet was combined with zinc oxide nanoparticles and deposited on the surface of polyurethane. The bactericidal activity against *E. coli*, *P. aeruginosa*, methicillin-resistant *S. aureus* (MRSA), and notably, highly resistant endospores of *Clostridioides* (*Clostridium*) difficile was reported.^170^

In the works of H. Bujdakova *et al.*, a hybird composed of polyurethane, layered silicate (saponite) and phloxine B^171^ or erythrosine B,^172^ showed effectiveness against *S. aureus*. The reduction of biofilm growth associated with the surface modification of PU was also observed under dark conditions.^171^

Organic photosensitizers can also be accompanied with gold or silver nanoparticles to boost the antimicrobial effect. A composite material comprising crystal violet, methylene blue, and nanometer-scale gold nanoparticles was applied as a coating on medical-grade silicone. The resultant material exhibited efficacy in the treatment of *S. epidermidis*, *S. cerevisiae*, MS2 Bacteriophage, *Pythium ultimum*, and the filamentous fungus *Botrytis cinerea.*^173^ In the work of Bryaskov *et al.*, bioinspired photoactive antibacterial polymer coatings on stainless steel were described. The photoactive coating, which is formed in a three-step deposition process involving a catechol-based primer for adhesion, a silver-doped nanogel for enhancing antibacterial properties, and a porphyrin-based photosensitizer that generates reactive oxygen species under visible light, showed effective antibacterial activity against *B. subtilis* and *E. coli*.^174^

**Fig. 17:**
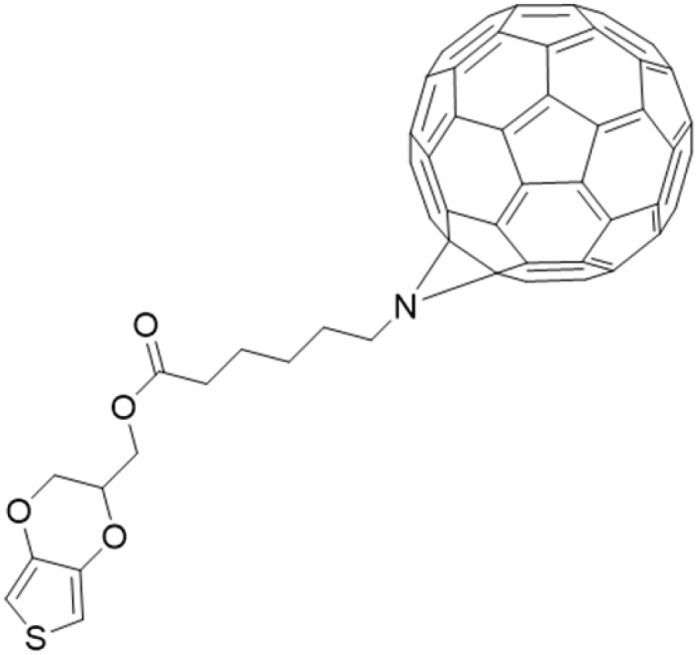
PEDOT-C60 investigated in (ref. 175).

Finally, organic photosensitizers hybrid with fullerene also show promising results. Two types of fullerene-containing organic layers were reported as light-activated antimicrobial layers. Lopez *et al.* reported the formation of PEDOT-fullerene C_60_ (Fig. 17) coating *via* electrochemical polymerization on ITO. The resulting layers inhibited *S. Aureus* by 99.9%.^175^ In the work of Ballatore *et al.*, a porphyrin-fullerene dyad substituted with carbazoyl units was electrodeposited on ITO to form a layer effective against *S. aureus* and *E. coli*.^176^

## Future outlook

**Fig. 18:**
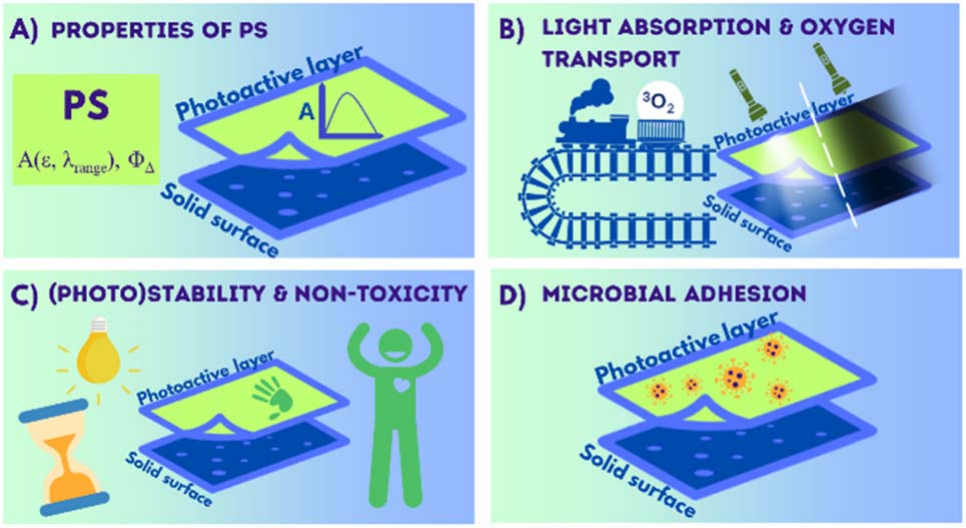
Summary of factors influencing the effectiveness of light-activated antibacterial coatings.

The significant progress in the design and application of the light-activated antimicrobial organic layers has been observed in recent years. A variety of techniques can be used for the deposition of layers containing organic photosensitizers on inanimate surfaces, including covalent and non-covalent immobilization of PS. Both, PS-only layers or those with additional components, can be produced. The early works employed mainly commercially-available well-known organic dyes. However, lately more sophisticated PSs with tailored properties are used. The non-covalent immobilization methods, *e.g.* drop-casting or dispersion in polymeric matrix, are usually more straightforward and less time-consuming than the covalent attachment of PSs. However, such layers are more likely to exhibit only short-term stability, due *e.g.* PS leakage. Though, great antimicrobial response is observed for many reported light-activated organic coatings, still several challenges need to be overcome prior to commercialization (Fig. 18).

### Properties of photosensitizer (PS)

The key part of light-activated antimicrobial layers is a photosensitizer that is responsible for ROS production and the ROS-related antimicrobial action. The rules for the selection or design of the proper PS are generally similar to classical PACT. Since the lower intensity of light affects the antimicrobial response, PS should possess strong absorbance in the UV-vis range (preferably broadband absorption), high efficiency of ROS production and high photostability (Fig. 18a).^177^ The formation of layers consisting of several PSs with complementary absorption in visible range^106,131,178^ can be considered as an alternative to the tedious synthesis of PS with various light-harvesting antennas. Additionally, the influence of a solid surface on the properties of deposited PS needs to be taken into account, since *e.g.* aggregation^132,179^ or change in the chemical structure of PS due to the involvement of functional groups in covalent bond formation,^146,178^ may strongly alter photophysical and photochemical properties of organic PS.

### Light absorption and oxygen transport

Light and oxygen are both crucial to initiate ROS formation. Thus, first of all, the PS's optical properties should be optimized, as described above. Second of all, the transport of light and oxygen/ROS within the layer has to be taken into account while designing light-activated layers (Fig. 18b).^134^ In the case of deposition of PS within polymeric matrices, polymers with high transparency and high oxygen permeability are preferable, *e.g.* PDMS.^180^ Moreover, the ROS produced by PS cannot be scavenged within/by the coating, since it would significantly lower its antimicrobial efficiency and may result in its degradation.

### (Photo)stability and non-toxicity of the layer

Low stability of the material is commonly a key factor limiting its practical use. When considering light-activated antimicrobial coatings, next to the photostability of the photosensitizer itself, the stability of the entire coating has to be considered: (i) its adhesion to the surface of an object, (ii) stability of other components of layer, especially against oxidation by ROS (Fig. 18c).^150^ The leakage of the photosensitizer should be avoided or should be minimal, to ensure the long-term effectiveness of the coating.^146,181,182^ Also, when the leakage of PS is observed, the exact contribution of the photoactive layer to the inactivation of microorganisms, is harder to assess, since the observed effect may be mostly due to PS being in the solution phase. Finally, the toxicity of the coating has to be assessed before the commercial use.

### Microbial adhesion

Another crucial aspect of the designed coating is the adhesion of microorganisms under dark conditions (Fig. 18d). The irreversible attachment of bacteria may lead to the formation of thick biofilm^183^ effectively limiting access to light.^150^ The adhesion of microorganisms depends on the properties of the surface, its roughness, hydrophobicity, charge, *etc.* The biofilm growth under dark conditions can be limited either by optimizing the anti-adhesive properties of the layer^159,171,183^ or by boosting so-called contact-killing properties,^127,146,158,168^*e.g.* by the introduction of quaternary ammonium groups.

## Summary

Light-activated layers provide a unique approach to deal with pathogenic microorganisms contaminating inanimate surfaces. Thanks to the production of ROS, such coatings usually show versatile and highly-effective antimicrobial action. Organic photosensitizers possess several advantages over inorganic ones, *e.g.* strong visible light absorption, high yields of ROS production, or tunability. Still, there is no straightforward and easily-scalable method for the deposition of durable organic photoactive coatings on a surface irrespective of its type. Thus, the practical use is generally limited to inorganic photoactive materials. The significant progress has been made in the research on organic photosensitizers and corresponding layers in the last years. Hence, we believe that there is still a great potential for organic PSs in light-activated antimicrobial coatings, either alone or in hybrid systems.

## Data availability

No primary research results, software or code have been included and no new data were generated or analysed as part of this review.

## Author contributions

K. S., I. G., P. M. – writing – original draft, visualization; A. B.-G. – supervision, writing – review & editing.

## Conflicts of interest

There are no conflicts to declare.
